# Chitosan and Its Nanoparticles: A Multifaceted Approach to Antibacterial Applications

**DOI:** 10.3390/nano15020126

**Published:** 2025-01-16

**Authors:** Emir Akdaşçi, Hatice Duman, Furkan Eker, Mikhael Bechelany, Sercan Karav

**Affiliations:** 1 Department of Molecular Biology and Genetics, Çanakkale Onsekiz Mart University, Çanakkale 17100, Türkiye; emirakdasci@stu.comu.edu.tr (E.A.); hatice.duman@comu.edu.tr (H.D.); furkan.eker@stu.comu.edu.tr (F.E.); 2 European Institute for Membranes (IEM), UMR-5635, University Montpellier, ENSCM, CNRS, Place Eugène Bataillon, CEDEX 5, F-34095 Montpellier, France; 3 Functional Materials Group, Gulf University for Science and Technology (GUST), Masjid Al Aqsa Street, Mubarak Al-Abdullah 32093, Kuwait

**Keywords:** chitosan, chitosan nanoparticles, antibacterial, drug delivery, agriculture, nanocomposites, physicochemical property, chitosan synthesis, food preservation

## Abstract

Chitosan, a multifaceted amino polysaccharide biopolymer derived from chitin, has extensive antibacterial efficacy against diverse pathogenic microorganisms, including both Gram-negative and Gram-positive bacteria, in addition to fungi. Over the course of the last several decades, chitosan nanoparticles (NPs), which are polymeric and bio-based, have garnered a great deal of interest as efficient antibacterial agents. This is mostly due to the fact that they are used in a wide variety of applications, including medical treatments, food, chemicals, and agricultural products. Within the context of the antibacterial mechanism of chitosan and chitosan NPs, we present a review that provides an overview of the synthesis methods, including novel procedures, and compiles the applications that have been developed in the field of biomedicine. These applications include wound healing, drug delivery, dental treatment, water purification, agriculture, and food preservation. In addition to this, we focus on the mechanisms of action and the factors that determine the antibacterial activity of chitosan and its derivatives. In conjunction with this line of inquiry, researchers are strongly urged to concentrate their efforts on developing novel and ground-breaking applications of chitosan NPs.

## 1. Introduction

**Figure 1:**
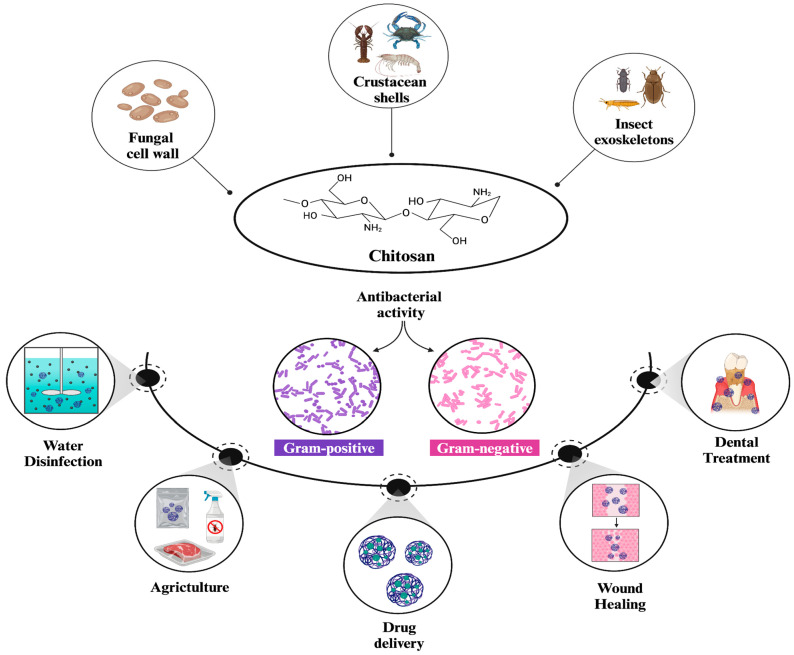
Structure and general applications of chitosan NPs [5,8]. Chitosan is naturally found in fungal cell walls, crustacean shells, and insect exoskeletons. It demonstrates potent antibacterial activity against both Gram-positive and Gram-negative strains of bacteria, which, in turn, enables its broader use in diverse applications such as water disinfection, drug delivery, wound healing, dental treatment, and agriculture.

Chitosan is a naturally occurring linear polysaccharide primarily found in crustacean shells, fungal cell walls, and insect exoskeletons [1]. It is obtained by the *N*-deacetylation of chitin, which is regarded as one of the most abundant polysaccharides in nature [2]. The structure of chitosan consists of alternating units of 1–4 linked *N*-acetylglucosamine (2-acetamido-2-deoxy-β-d-glucopyranose) and glucosamine (2-amino-2-deoxy-glucopyranose), (Figure 1) [3]. Depending on their molecular weight, chitosans are categorized as high molecular weight (HMW) or low molecular weight (LMW), which significantly influences their properties and preferred application areas. For instance, LMW chitosans are highlighted by their high solubility, biological activity, and capability to initiate interactions compared to HMW chitosans [4]. The degree of deacetylation (DA) is also an important factor that affects both the structure and properties of chitosan. A higher degree of deacetylation (DA) correlates with increased water solubility and biological activity, similar to LMW chitosans [5]. Additionally, an increased DA) can reduce both adhesion and cohesion forces in HMW chitosans, while it primarily reduces adhesion forces in LMW chitosans [6]. Moreover, the structural characteristics of chitosans include three reactive functional groups, a primary amine, and primary and secondary hydroxyl groups. These groups enable the production of polymers with unique properties and behaviors for various biomedical applications by facilitating a number of modifications [7].

The utilization of chitosan has emerged as one of the cutting-edge topics in polymer science, as it significantly contributes to the development of novel, cost-effective, and eco-friendly approaches [9]. The main reason for this is the advantageous characteristics of chitosan, such as non-toxicity, biodegradability, and biocompatibility, stemming from its inherent nature [10].

Currently, the ongoing research in the literature highlights chitosan as a potent antimicrobial agent [11,12]. Among the antimicrobial properties, bactericidal efficiency is the most expressed and studied characteristic of chitosan.

To give a few examples, Rani *et al.* highlighted the antibacterial activity of chitosan, derived from the shell of *Cherax quadricarinatus,* against both Gram-positive *Staphylococcus aureus* (*S. aureus*) and Gram-negative *Escherichia coli* (*E. coli*) [13]. From another perspective, Wang *et al.* evaluated the antibacterial characteristics of liposomes decorated with varying concentrations of chitosan (0, 0.25, 0.5, 1, 2, 3, and 4 mg/mL). The results revealed rapid and long-term bactericidal efficiency, with increasing concentrations leading to stronger outcomes, as evidenced by lower minimum inhibitory concentration (MIC) values [14].

Apart from their employment in the natural form, there is also growing interest in chitosan NPs in the field of nanotechnology [15]. NPs are nanostructures with sizes ranging from 1 to 100 nm [16]. They possess unique characteristics due to their high surface-area-to-volume ratio, tunable physicochemical properties, and ease of functionalization with various substances. Moreover, NPs can be tailored specifically for desired uses, from antibacterial studies to the development of biosensors, depending on their synthesis methods [17,18]. These methods include physical, chemical, and biological methods, each with distinct advantages and disadvantages [19].

Currently, researchers are mostly focused on the development of nature-friendly green synthesis approaches in order to overcome toxicity, which is regarded as one of the major limitations associated with commonly employed nanomaterials, such as silver and gold NPs [20]. Chitosan NPs, in this aspect, can be considered a potent candidate to replace these metallic NPs, being a naturally occurring, biodegradable, and cost-effective compound.

Chitosan NPs have wide-ranging applications in the current literature, such as cancer diagnosis, drug delivery, antioxidant therapy, antimicrobial therapy, water treatment, agriculture, and so on [15]. Among these applications, the antibacterial properties of chitosan NPs are among the most extensively studied and widely utilized due to their strong research background and practical applications. Chitosan NPs exhibit multiple antibacterial mechanisms against a wide range of bacteria [15]. They can initiate electrostatic interactions with negatively charged bacterial residues and interfere with cellular pathways, thereby preventing proliferation and inducing cell death [21]. As a result, studies on harnessing the superior antibacterial activity of chitosan NPs have been ongoing for many years.

Furthermore, both chitosan and chitosan NPs have been combined with various materials to develop bactericidal nanocomplexes for biomedical applications [22,23]. Similar to the antibacterial mechanism of chitosan NPs, chitosan-based nanocomplexes and hybrid particles can directly interact with bacterial cell walls and cellular components, disrupt intracellular synthesis pathways, inhibit bacterial growth, and induce cell death [24]. Additionally, the antibacterial activity of chitosan NPs is heavily influenced by physicochemical and environmental factors, such as pH, temperature, medium composition, particle size, capping agents, and molecular weight [21]. These properties also influence the composition and activity of chitosan-based nanostructures, making both the synthesis process and structural variations critical considerations for their applications. These factors are discussed in detail in the following sections.

Beyond biomedicine, the use of chitosan also extends to agricultural applications, where antibacterial food packaging and food preservation materials are produced [25,26]. Many chitosan films, including various materials such as metal ions (e.g., silver, titanium dioxide, and copper), nanocrystals, NPs, and extracts, have been widely applied in food packaging to provide antibacterial activity, enhance thermal stability and mechanical properties, and extend the shelf life of various products [27]. Additionally, chitosan NPs are commonly incorporated into nanocomposites, further advancing the development of food packaging materials. However, the incorporation of chitosan NPs significantly affects both the characteristics and functional capabilities of composites, making it an option that requires detailed consideration rather than direct application [28].

Similar to their applications in food packaging, chitosan NPs show significant promise in dentistry. Many chitosan-based biomaterials have been used in dentistry for similar reasons [29]. In addition, chitosan NPs have significant potential in dental caries treatment, serve as a key component in developing delivery systems for periodontal disease therapies, and are combined with various materials for implants [30].

Another promising strategy to utilize the antibacterial activity of chitosan NPs is the wound healing applications. The use of nanochitosan and its derivatives, either alone or in combination with different molecules, holds great importance for advancing current wound-healing studies, as they promote the acceleration of the healing process [31]. The main reason for this is the immense antibacterial activity of chitosan, which enables the development of non-toxic, biodegradable, and environmentally safe dressings for proper wound care management. In addition, owing to their non-allergenic nature, various research utilizing chitosan-based nanomaterials have been conducted *in vivo* to optimize and facilitate their wide-scale application in the future [32,33,34].

Moreover, researchers have been employing chitosan NPs in industrial areas for water purification due to their high efficiency in the removal of pollutants, such as heavy metal ions and commercial dyes [35]. A notable application of chitosan NPs is the disinfection of bacteria from water, in which positively charged functional groups of chitosan NPs interact with negatively charged cell membranes to exert antibacterial effects [36]. Collectively, these results not only position chitosan NPs as promising candidates for industrial applications but also offer cost-effective solutions for addressing challenges. Hence, optimization and improvement of important parameters affecting this process would be crucial to maximize the efficiency of the synthesized chitosan NPs for further use.

In this review, we have delved into antibacterial studies of chitosan and chitosan NPs by focusing on recent advances. We have emphasized not only the general antibacterial characteristics of chitosan but also discussed the factors influencing these characteristics, with a particular focus on the synthesis methods and physicochemical properties. Moreover, we highlight the role of physicochemical properties when chitosan and its particles are incorporated into various nanomaterials, focusing on how their antibacterial applications can be affected.

Since chitosan has recently become one of the most favored materials in nanotechnology, owing to its eco-friendly nature and superior characteristics, multiple studies have been published regarding its antibacterial applications. Considering this, we have further discussed the current status of chitosan in nanomaterial science by providing a literature analysis of the published documents related to both chitosan and chitosan NPs in the last five years.

This review aims to present researchers with the current status of antibacterial applications of chitosan and its incorporation into various nanomaterials, emphasizing the influence of physicochemical properties on antibacterial mechanisms and highlighting areas where chitosan shows significant potential, such as agriculture, food safety, drug delivery, and water purification.

## 2. Synthesis of Chitosan Nanoparticles

Chitosan NPs were initially described by Ohya *et al.* for the systemic administration of 5-fluorouracil, a chemotherapeutic agent [37]. Using emulsification, precipitation, or crosslinking, researchers have developed techniques for generating chitosan NPs based on variables, such as size, stability, drug-loading capacity, and retention period [38]. The earliest technique for producing chitosan NPs that was documented in the literature used emulsification and crosslinking, which combined the aldehyde group of a crosslinking agent with the amino group of chitosan [37].

The emulsification solvent diffusion method was first described by El-Shabouri [39]. The Poly(lactic-co-glycolic acid) (PLGA)-based approach was developed by Niwa *et al.* and is a modified version of this method [40]. The formation of an emulsion is accomplished by first infusing an organic phase into a chitosan solution that contains a stabilizing agent, such as poloxamer, and then homogenizing the mixture under high pressure. After this, the emulsion is diluted with water, which results in the simultaneous precipitation of polymers and the production of NPs [15].

Another approach that is based on covalent crosslinking is the reversed micelle (microemulsion) method. This method is comparable to emulsification and crosslinking in that it is a microemulsion. In order to produce chitosan NPs, the reverse micellar procedure requires dissolving a surfactant in *N*-hexane and chitosan in an acetic solution, then adding glutaraldehyde, agitating the mixture at room temperature, and finally creating tiny particles. During the course of one night, the process of crosslinking between the free amine group of chitosan and glutaraldehyde is completed. When used in this procedure, glutaraldehyde performs the function of a crosslinker. Next, the organic solvent is eliminated by the process of evaporation under low pressure. Following this, the surplus surfactant was eliminated through the precipitation of CaCl_2_ and the subsequent removal of the precipitant using centrifugation [41]. Reverse micellar methods eliminate the need for crosslinkers and highly hazardous chemical solvents, resulting in the production of ultrafine NPs with a restricted size range. In order to do this, chitosan is added to an organic solvent that contains a surfactant, and then reverse micelles are formed while the mixture is continuously agitated [42]. Using this method, one of the most important characteristics for a wide variety of applications in which the specific surface area plays a role is the fact that it is feasible to produce ultrafine NPs with a size less than 100 nm [43].

Emulsification and precipitation are fundamental components of the phase inversion precipitation technique. Chitosan NPs may also be synthesized using precipitation-based techniques. The utilization of emulsification along with precipitation is an essential component of the phase inversion precipitation technique. An organic phase consisting of dichloromethane and acetone, together with an aqueous solution of chitosan, is used in the process of creating the oil-in-water emulsion in the presence of a stabilizer known as a polyoxamer. During the process of high-pressure homogenization, nanometer-sized emulsion droplets are produced. These droplets are then separated by evaporation at low pressure and room temperature. This process leads to the diffusion of acetone out of the droplets and the precipitation of NPs [39]. A different approach, known as desolvation or emulsion-droplet coalescence, has been reported. It is based on the coalescence of two water-in-oil emulsions, which causes NPs to precipitate since one of the emulsions contains NaOH, which acts as a precipitation agent. Two emulsions are prepared using sorbitan sesquioleate with liquid paraffin, chitosan, and NaOH. The chitosan emulsion is prepared via high-speed homogenization. As NaOH diffuses into ultrafine droplets, it reduces the solubility of chitosan, which causes precipitation and production of NPs. The processes of centrifugation, solvent washing, and freeze-drying are used to produce NPs [44]. Thus, precipitation techniques produce NPs with sizes greater than 600–800 nm; however, these techniques are rarely chosen since they involve the use of organic solvents and demand a high level of energy homogeneity. In spite of the limited amount of research that has been conducted in the literature, the phase inversion precipitation approach is able to produce chitosan NPs that have high encapsulation effectiveness for hydrophobic medicines such as Cyclosporin A [45].

The ionotropic gelation method is another effective approach for the synthesis of chitosan NPs. An electrostatic contact takes place between the amine group of chitosan and a negatively charged polyanion such as tripolyphosphate (TPP) in order to bring about the desired effect. In the presence of acetic acid, chitosan can dissolve without the need for stabilizing chemicals such as poloxamers. The addition of polyanion results in the spontaneous formation of NPs while the mixture is being stirred mechanically at room temperature. Through the manipulation of the proportion of chitosan in the stabilizer, it is possible to alter the size of the particles as well as their surface charge. It was discovered that there was a general increase in particle compactness and size when the concentration of chitosan was increased, as well as when the ratio of polymer to polyanion was increased [46]. This method was initially described by Calvo *et al.*, and since then, it has been subjected to much research and development [47]. Through this approach, insulin, silk peptide, and serum albumin have all been effectively administered through the oral route. A restricted number of applications are now accessible, perhaps attributable to an extended production process [45,48].

It is also possible to utilize ionic gelation in conjunction with radical polymerization, which causes the gelation of chitosan to occur concurrently with the polymerization of acrylic or methacrylic acid [49]. Potassium persulfate is used as an initiator in the polymerization procedure, which necessitates a stirring period of 6 h at temperatures ranging from 60 to 70 °C [50]. Oral administration of insulin, silk peptide, and serum albumin has been effectively accomplished via this approach. Apparently, as a result of the lengthy development process, a few apps are now accessible [45,48].

Self-assembly is a frequently used process that is based on many simultaneous contacts between chitosan and other molecules. These interactions can be electrostatic, hydrophobic, or linked by hydrogen bonding or van der Waals forces. NP synthesis can occur by self-assembly, which is a method that is extensively utilized [51]. By agitating polymer solutions, chitosan polyelectrolytes may form complexes with naturally occurring anionic substances, such as alginate or hyaluronic acid. With acyl-chitosan, stearic acid-grafted chitosan, and PEGylated chitosan affecting the hydrophobic interactions during self-assembly, the hydrophobicity may be altered by grafting. When it comes to encapsulating hydrophilic and lipophilic pharmaceuticals, NPs that are generated by self-assembly are particularly advantageous. This is because they enable the active ingredient to remain stable within the biocompatible matrix, which can be easily changed using this gentle process.

One common technique used in the fabrication of chitosan NPs is the conjugation of chitosan with various polymers through a free radical reaction. Free radical grafting is a chemical process used to create chitosan-polyphenol conjugates. The reaction involves the formation of chitosan radicals within a redox system that includes hydroxyl radicals, enabling polymers such as polyphenols to covalently bond to chitosan radicals at multiple positions [52]. As discussed in later sections, polymer modification of chitosan presents a promising alternative for addressing various applications and persistent challenges. Polyphenol-incorporated chitosan materials synthesized using the free radical technique represent a common and efficient approach for various applications, particularly in agricultural sectors such as food packaging [53]. The primary reason behind this application is that this functionalization can notably increase the antibacterial capacity of chitosan [54]. Such techniques during chitosan NP fabrication are very important for synthesizing functionalized particles.

The polyelectrolyte complex (PEC) approach entails the synthesis of chitosan NPs by the incorporation of oppositely charged polymers or counter ions into a chitosan solution. Chitosan is often used in conjunction with acetic acid in order to dissolve the oppositely charged polymer or counter ion. This process is carried out under ambient conditions while stirring [39]. The formation of the PEC formulation takes place as a consequence of the electrostatic interaction that takes place between the chitosan, which is anti-charged, and the extra polymer or counter ion, which ultimately leads to the neutralization of charge. Therefore, as a result of the neutralization of charges, the PEC is able to self-assemble, which results in a significant decrease in its hydrophilicity ability. It was stated that the NPs that were produced by this method ranged in size from 50 to 700 nm [55]. The researchers Sharma *et al.* came to the conclusion that the IgA-loaded chitosan–dextran nanoparticles that were generated by the PEC process offer a straightforward and efficient approach to the development of a drug delivery system [56].

The electrospraying method is a relatively new method that is currently being used to generate NPs from biopolymers [57]. Electrospray is a variation of the electrospinning technique, which has been used in medicine for the purpose of drug encapsulation. This technique is currently expanding into other sectors, particularly food technology, due to its simplicity and the utilization of inexpensive equipment. There are two types of electrospray techniques based on the method of droplet collection: electrospray in plates and electrospray in solution [58]. The electrospray principle relies on the capacity of an electric field to distort the droplet interface, producing droplets in the micrometer or nanoscale range, contingent on the controlling parameters. Despite the simplicity of the electrospray approach, it is essential to consider the control factors to obtain optimal polymer particles in terms of particle size and size distribution. These variables encompass system characteristics, including the solution flow rate, applied electric potential, collector distance, solution viscosity, conductivity, surface tension, molecular weight, and polymer concentration [59].

The research on chitosan nanostructures is substantially less extensive than that conducted using bottom-up strategies, as the “top-down” approach in nanofabrication entails disintegrating a larger parent superstructure to create nanostructures [60]. To manufacture NPs using the top-down method, chitin is first hydrolyzed with acid to produce chitin nanocrystals, and then deacetylation is performed to replace the acetyl group with an amino group. Centrifugation and washing are two of the procedures involved in this process. Hydrochloric acid is used to disrupt glycosidic bonds, and the amorphous component is removed. As a result of many centrifugation stages, chitin nanocrystals are successfully separated. In order to achieve chitosan NPs with a degree of deacetylation of more than 60%, alkaline treatment is used for the deacetylation process [60].

On the other hand, chitosan extraction by chemical processes has a number of disadvantages, including the fact that it can alter physicochemical qualities, result in the presence of chemicals in wastewater effluents, and lead to an increase in purification costs. As a result, biological/green synthesis methods have become increasingly popular.

The biological technique utilizing microorganisms proved superior to the chemical method, as it maintained the structural integrity of chitin. Spray drying is one of these approaches; chitosan is mostly dissolved in aqueous acetic acid, and NPs are produced by passing this solution through a nozzle at temperatures ranging from 120 to 150 °C. This technique is used rather frequently in the manufacture of chitosan microparticles as well as in the separation of NPs that have been acquired via the use of other techniques [50]. The supercritical-CO_2_-assisted solubilization and atomization (SCASA) technique is an environmentally friendly technology that eliminates the need for toxic solvents by being prepared simply using water and carbon dioxide. Chitosan is dissolved in water by means of compressed carbon dioxide under high pressure, which is a pioneering environmentally friendly technology. The chitosan solution is delivered to a fluidized bed by a spraying nozzle, which ultimately results in atomization after a dissolving stage that takes a considerable amount of time (48 h). In the course of the drying process, NPs are produced, and they are collected by a filter that is situated on top of the fluidized bed [61].

**Figure 2:**
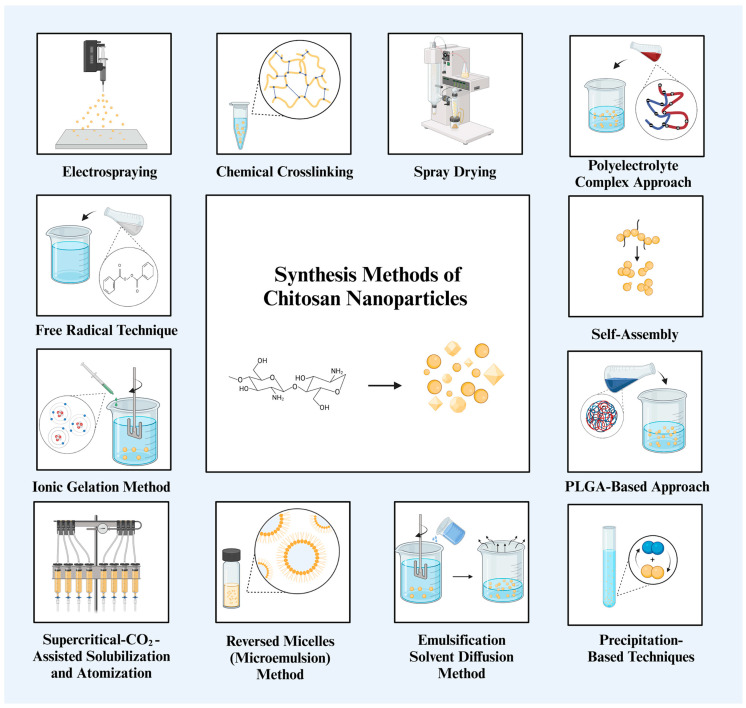
General summary of chitosan NP synthesis methods [15,45]. Chitosan can be synthesized into NPs with various shapes, spherical, triangular, and cubic, through a variety of synthesis methods.

The overall assessment of the preparation techniques is shown in Table 1 and Figure 2, along with the benefits and limitations of each approach with regard to NP properties, hazardous chemical usage, and simplicity of preparation. Methods that follow mild processes and generate NPs quickly, such as ionic gelation, self-assembly, and spray drying, appear to be the most important choices from the perspective of human health and a sustainable future, although it is impossible to single out one technique or principle as the best for all applications.

## 3. Antibacterial Mechanism of Chitosan Nanoparticles

Bacterial resistance has become a significant threat to humanity over the past 25 years, and it may soon reach a point where even minor illnesses can become fatal. There is an urgent need for the discovery and production of new and more powerful antimicrobial compounds because of the rise in multidrug-resistant bacteria and the dearth of new antimicrobial medications on the market. NPs based on chitosan have demonstrated great promise as antibacterial agents [66].

Chitosan and its derivatives have garnered a lot of attention because of their antibacterial properties. In actuality, chitosan’s antimicrobial properties are advantageous for a variety of commercial uses, such as food preservation, production of wound dressings, and antimicrobial-finished fabrics [67]. Numerous parameters, such as the type of chitosan, degree of polymerization, and some of its other physicochemical characteristics, affect its antibacterial effectiveness [50]. Compared to Gram-negative bacteria, chitosan has a stronger antibacterial activity against Gram-positive bacteria. Additionally, influenced by solvent and molecular weight, chitosan’s antibacterial activity is negatively correlated with pH, being more active at lower pH levels [68,69].

Because of their small dimensions and quantum size impact, NPs have a unique property that may allow chitosan NPs to show better activities. The mechanism of chitosan NPs’ antibacterial action has been explained by a number of theories, most likely involving communication with the bacterial cell wall or membrane. The electrostatic interaction between the positively charged amino groups of glucosamine and negatively charged bacterial cell membranes is the most well-known chitosan NP mechanism of antimicrobial activity [70]. This contact triggers significant alterations in the cell surface, resulting in changes in membrane permeability that subsequently provoke osmotic imbalance and the outflow of intracellular chemicals, culminating in cell death [67,71,72].

The electrostatic force between chitosan and the bacterial cell wall facilitates tight contact with charged molecules, resulting in the penetration of chitosan NPs into the bacterial cell wall [73]. Thus, the likelihood of chitosan NPs collecting at the site of contact escalates. Furthermore, chitosan NPs can alter the electron transport pathway of bacteria. The principal antibacterial mechanisms of chitosan include electrostatic interactions, modification of membrane permeability, DNA binding, and disruption of DNA replication, resulting in bacterial cell death. Its reduced molecular weight enables cellular entry and inhibits the replication machinery. The flocculation of electronegative components by chitosan within the cell disrupts the physiological functions of bacteria, resulting in bacterial cell death [74].

**Figure 3:**
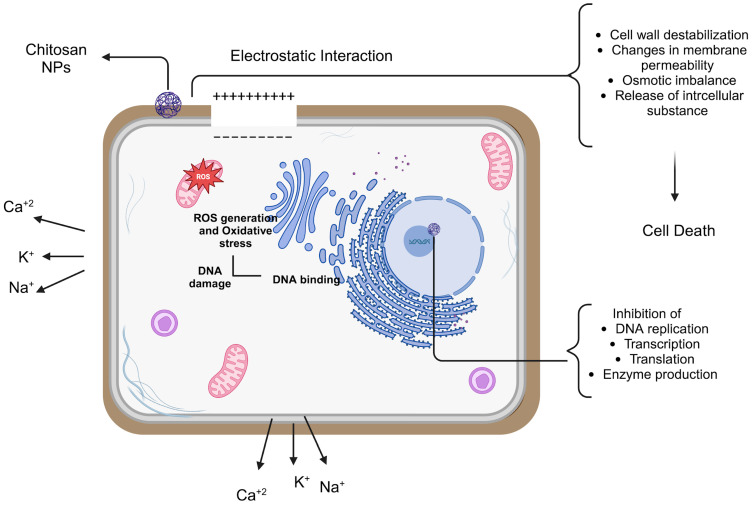
Antibacterial mechanisms of chitosan NPs, different concentrations of chitosan, and TPP. The chitosan NPs prepared with 0.1% TPP and 0.25% chitosan showed effective antibacterial activity against *Pseudomonas aeruginosa* (*P. aeruginosa*) and *S. aureus* [76]. The antibacterial activity of chitosan NPs against *E. coli* and *S. aureus* was examined in a related investigation. In contrast to chitosan NPs without TPP, which showed reduced activity, chitosan NPs with TPP demonstrated strong suppression of both *E. coli* and *S. aureus* [77].

A probable mechanism is the chelating activity of chitosan against metal ions, which promotes toxin synthesis and inhibits bacterial survival. Chitosan has antibacterial effects owing to its capacity to chelate metal ions, including Fe^2+^, Mg^2+^, Ni^2+^, Co^2+^, Cu^2+^, and Zn^2+^, in acidic circumstances. This method is most efficient at elevated pH when chitosan captures positive ions owing to the unprotonated NH_2_ groups and available electron pairs on the amine nitrogen. Chitosan molecules can obstruct essential nutrition transport, resulting in cell death, therefore necessitating careful consideration of several parameters for the effective application of chitosan NPs (Figure 3) [75].

Derivatives of chitosan have strong antibacterial activity against a range of bacterial species. Chitosan NPs derived from a chitosan derivative, namely betaine, were evaluated for antibacterial efficacy. Chitosan compounds with medium molecular weights and elevated degrees of substitution showed superior antibacterial efficacy compared to commercial antibiotics. These findings indicate that a higher degree of substitution results in enhanced antibacterial activity of chitosan with varying molecular weights [78]. Compared to pure chitosan, chitosan NPs derived from the most potent heterocyclic derivative of chitosan with a modest molecular mass increased the antibacterial activity by around three times [79]. A study described a novel approach for the manufacturing of chitosan NPs that substitutes chemical crosslinking with cinnamaldehyde for the conventional technique of utilizing TPP as an ionic crosslinker. The inhibitory effect of chitosan was considerably enhanced from 62% to 96% against *E. coli* and from 65% to 98% against *S. aureus*, indicating a synergistic antibacterial action [80].

Thus, the antibacterial properties of chitosan and its derivatives are both safe and efficient. The goal of recent research has been to create more powerful chemicals by decreasing and encapsulating metal NPs. In order to synthesize NPs from natural resources, such as plant and bacterial extracts, new methods are being developed. Chitosan NPs may work in concert to provide antibacterial activity, which might result in the development of a new class of antimicrobial drugs.

## 4. Effect of Physicochemical Properties of Chitosan and Chitosan NPs in Antibacterial Applications

**Figure 4:**
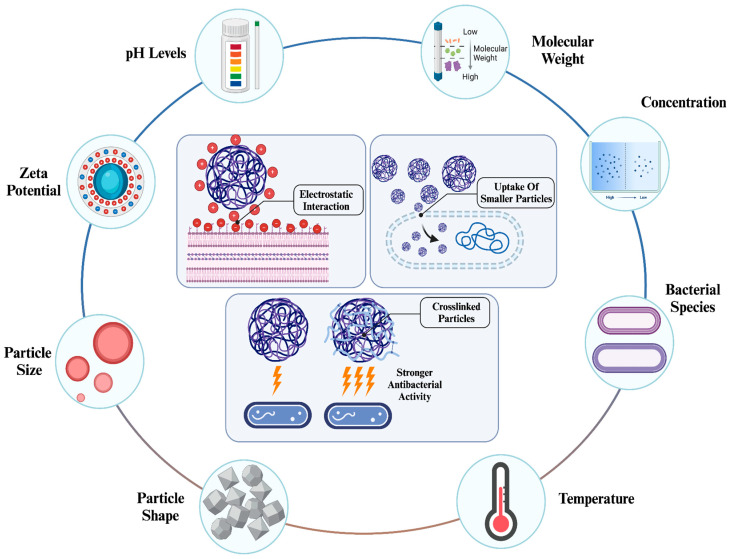
Factors influencing antibacterial activity of chitosan NPs [86]. The antibacterial activity of chitosan NPs is significantly influenced by their physicochemical properties, environmental conditions, and type of bacterial species. Depending on these conditions, chitosan NPs can initiate electrostatic interactions due to their high zeta potential, enhance internalization through smaller sizes, and exhibit stronger activity with crosslinking.

The physicochemical properties of NPs are major factors influencing their activity. It has been emphasized that properties such as size, shape, surface charge, and optical characteristics greatly alter the efficiency of NPs in various applications [81,82]. Among these applications, the efficiency of antibacterial therapy can be significantly influenced by the properties of NPs [83,84]. For instance, physicochemical properties significantly impact the cellular uptake of NPs. Depending on the type of NPs, various properties can increase or reduce the uptake efficiency. Generally, small-sized NPs can be taken up more easily, surface charge can enhance the initiation of cellular interaction, surface modification with various biomolecules can increase both specificity and uptake efficiency, and certain shapes (such as spherical) lead to more efficient uptake compared to their counterparts (Figure 4) [85].

These properties not only affect the applicability of NPs in nanomedicine but also ensure their safety through controller synthesis and determined properties [87]. This is why, like other types of NPs, the physicochemical properties of chitosan NPs need to be considered to achieve high efficiency in antibacterial therapies and prevent any adverse effects the NPs may cause (Table 2). The changes in the properties of chitosan NPs can affect their antibacterial capability in therapies.

### 4.1. Effect of Surface Chemistry of Chitosan NPs in Their Antibacterial Applications

Surface chemistry is another crucial factor influencing the application efficiency of NPs. Surface modification of NPs modifies their charge density and biological characteristics. Depending on the charge density, NPs can exhibit the following features: naturally charged particles enhance stability while minimizing direct interaction with biological systems (increasing circulation), positively charged particles promote interactions with anionic residues on cell surfaces, and negatively charged particles influence aggregation and cellular uptake mechanisms [95]. Various approaches for the surface modification of NPs include covalent conjugation, noncovalent functionalization, polymer functionalization, and cross-coupling [96]. Some of these methods have been applied to chitosan NPs to enhance their applications. For instance, the surface chemistry of chitosan NPs has been modified using crosslinking in multiple studies to increase the efficiency and stabilize the particles in various applications, particularly in the development of drug delivery systems for various areas [97,98]. Drug delivery is one of the most common approaches that is used in chitosan NP-based antibacterial therapy. The usage of chitosan NPs as both an antibacterial agent and a carrier for other antibacterial agents is significantly promising in antibacterial therapies. Therefore, research has been conducted to improve chitosan NP-based antibacterial therapies by enhancing both its carrier capability and antibacterial activity.

#### 4.1.1. Effect of Crosslinking

Crosslinking alters the surface chemistry of chitosan NPs, which may impact their antibacterial application.

TPP is one of the most common crosslinking agents used in biomedical applications of chitosan NPs, including antibacterial therapy and drug delivery [99].

During synthesis, certain factors influence the physicochemical properties of chitosan NPs during crosslinking. As an example of TPP, the concentration of both chitosan and TPP, pH of the solution, and salinity can impact the physicochemical properties of the final product [100]. Moreover, the crosslinking degree might also change the activity of the particle. This was evaluated in a study that demonstrated the effect of the degree of TPP crosslinking on both the properties and antibacterial capability of chitosan NPs [101].

Khoerunnisa *et al.* investigated the physicochemical properties of TPP crosslinked chitosan NPs with antibacterial activity [102]. The experiments emphasized the following changes in the physicochemical properties of TPP-chitosan NPs. The particle size was inversely proportional to the chitosan concentration, with the highest concentration (2%) resulting in the smallest average particle size of 79.244 nm. SEM images revealed slight structural changes depending on the chitosan concentration; lower concentrations produced smooth, sheet-like particles, while higher concentrations resulted in small chunks with smoother surfaces and homogenous sizes. Moreover, the most significant changes in the particle morphology were attributed to the degree of crosslinking. TPP crosslinking caused a blue shift in the surface plasmon resonance (SPR) peak of chitosan from 234 nm to 231–228 nm. Changes in antibacterial activity were observed in *S. aureus* and *E. coli*. The zone of inhibition (ZOI) of TPP crosslinked chitosan NPs was up to 2-fold higher than that of unlinked chitosan at the highest chitosan concentration (2.5 ± 0.15, 2.3 ± 0.15, 5.5 ± 0.27, and 5 ± 0.25, respectively). The increased charge density of the chitosan NPs achieved through TPP crosslinking enhanced their bactericidal activity. The most important finding was the enhanced antibacterial activity of TPP-chitosan NPs against both Gram-positive and Gram-negative bacteria. Gram-negative bacteria possess negatively charged cellular surfaces, facilitating chitosan NP uptake via a positive charge density, whereas Gram-positive bacteria lack this feature, suggesting an alternative mechanism for antibacterial activity. Researchers have emphasized the role of lipoteichoic acid in initiating chitosan interactions.

Considering the proven drug-carrying capability of chitosan NPs, they have been widely utilized in various antibacterial drug delivery applications. Surface modification is an efficient method that is widely employed in the development of numerous drug delivery systems, including those incorporating chitosan NPs. Crosslinking can enhance the drug encapsulation efficiency and release profile of chitosan NPs [103]. As drug delivery constitutes a significant portion of the antibacterial applications of chitosan NPs, the impact of physicochemical properties on these systems must be discussed to emphasize the role of surface modification in this field.

Depending on the crosslinking agent, the drug delivery capability of chitosan NPs significantly changes, which may alter their efficiency in antibacterial applications. As an example, a similar experiment was conducted to investigate the effect of various crosslinking agents on the properties and drug release profile of chitosan NPs [104]. In a comparison of three different crosslinking agents, TPP, phytic acid (PA), and sodium hexametaphosphate (SHMP), several properties of the system were observed. While there were no significant changes in the morphological characteristics, their size greatly differed based on the capping agents after the encapsulation of myricetin. TPP crosslinked chitosan NPs demonstrated the smallest size, 146.1 ± 11.3 nm, and the encapsulation efficiency, 30.1 ± 0.7%, while both of these values were approximately 50% higher for the other two agents (at pH value of 3). Conversely, when the pH value increased to 5, TPP crosslinking became the most efficient agent with the highest 47.4 ± 0.2% encapsulation efficiency and the smallest size of 183.6 ± 0.4 nm. Drug release behavior was constant and slower for SHMP and PA, while it was approximately 2-fold higher for TPP. The effect of the agents on mucoadhesiveness was also observed, with PA crosslinking showing superiority over the other two agents. These results now only demonstrate the impact of crosslinking agents on delivery efficiency, but also the factors during the parameters, such as pH and mucoadhesivity, can alter the efficiency of these agents for specific applications. Similar studies have investigated the change in drug delivery capability of chitosan NPs by comparing crosslinking agents, including other types of nanocomplexes [98].

As an example of antibacterial drug delivery, Nayak *et al.* demonstrated crosslinked chitosan NPs with tannic acid (TA) and borax acid (BA) to deliver metronidazole against bacterial vaginosis [105]. The choice of crosslinking agent significantly influenced the size and zeta potential of the particles before drug loading. TA crosslinking resulted in particles with a size of 256.06 ± 6.5 nm and a zeta potential of 36 ± 2.1 +mV, whereas BX crosslinking produced larger particles measuring 341.36 ± 6.2 nm and a zeta potential of 45 ± 3.1 +mV. The particle size difference exceeded two-fold, with TA-chitosan NPs measuring 171.96 ± 7.2 nm compared to 380.16 ± 8.4 nm for BA-chitosan NPs. However, the disparity in the zeta potentials was less notable. TPP was employed as a third crosslinking agent and was included in subsequent experiments. The antimicrobial activity of the particles was evaluated through *in vitro* experiments involving *E. coli* and *Candida albicans* (*C. albicans*). The MIC values against *E. coli* were as follows: for BX crosslinking, 79 ± 0.7 μg/mL (unloaded) and 24 ± 0.6 μg/mL (loaded); for TA crosslinking, 48 ± 0.3 μg/mL (unloaded) and 32 ± 0.4 μg/mL (loaded); and for TPP crosslinking, 161 ± 0.7 μg/mL (unloaded) and 158 ± 0.3 μg/mL (loaded). Interestingly, TA-crosslinked chitosan NPs exhibited the strongest antibacterial activity without drug encapsulation, whereas BX crosslinking achieved the lowest MIC value with drug encapsulation (61.5 ± 1.06 encapsulation efficiency), demonstrating the highest antibacterial activity in the experiment. The biofilm quantification assay and *in vivo* antibacterial activity supported the significant activity of drug-loaded BX-chitosan NPs.

Based on the discussed studies, crosslinking chitosan NPs not only modifies their physicochemical properties but also significantly impacts their antibacterial activity and drug-carrying capability. Surface modification through crosslinking is a promising approach for enhancing antibacterial applications. However, alterations in properties and activities require thorough investigation to identify the most effective approach.

#### 4.1.2. Effect of Surface Charge Density

Another important factor that affects the antibacterial activity of NPs is their charge density. Depending on the charge density, either anionic or cationic, NPs can initiate stronger interactions with bacterial cells, leading to potent mitochondrial damage and higher cellular uptake [106]. Chitosan NPs are known to enhance cellular uptake when they have positive charge density, which was shown against several types of cell lines. Similar to other properties, factors during the synthesis process, especially pH, have a notable impact on the determination of the surface charge density and zeta potential of chitosan NPs.

Their surface charge is altered during the synthesis process of the particles. Athavale *et al.* reported the tunable surface charge of chitosan NPs within a pH range of 2–9 [107]. It was emphasized that chitosan NPs demonstrated a more stabilized nature and higher zeta potential compared to particles that are found in natural and basic environments. While the chitosan NPs had a zeta potential of approximately 42 mV in the most acidic environment (2 pH), this value almost reached 0 mV at a pH near 9. In addition, when the pH levels exceeded 5, the NPs showed a high rate of aggregation. It can be concluded that positively charged chitosan NPs can exhibit high stability and affinity towards negatively charged residues, which will make their antibacterial mechanisms more precise.

Chang *et al.* demonstrated the influence of pH values and molecular weight of chitosans on the zeta potential and antibacterial activity of chitosan [108]. Chitosans with molecular weights ranging from 3.3 to 300 kDa were synthesized at both acidic and neutral pH levels. Depending on the temperature and pH levels, chitosan exhibited the lowest molecular weight of 3.3 kDa at pH 7 and demonstrated the strongest antibacterial activity during the experiments. However, at acidic pH levels, the antibacterial activity of chitosans was proportional to their molecular weight, which was the opposite at neutral pH levels. Changes in the zeta potential also varied depending on the pH level. At acidic pH levels, an increase in molecular weight corresponded to a proportional increase in zeta potential, whereas a lower molecular weight resulted in a higher zeta potential. In contrast, at neutral pH levels, an increase in molecular weight significantly reduced the zeta potential to negative values, thereby affecting antibacterial activity. These factors are crucial for the synthesis of chitosan NP to control their antibacterial activity. In this case, a similar study used chitosan NPs, where the smallest chitosan NPs with the highest zeta potential demonstrated the highest activity [109].

However, it should be mentioned that there are certain cases where negatively charged chitosan NPs possess potential utilization for certain applications, such as in the development of drug delivery systems [110]. However, their affinity to aggregate in a non-positive charge density should be carefully considered, as it can affect their involvement in drug delivery applications, where they tend to be used in nanocomplexes such as nanogels [111].

#### 4.1.3. Polymer Conjugation and Delivery in Enhancing Antibacterial Application of Chitosan NPs

As highlighted in the previous paragraphs, surface modification is one of the most effective and widely preferred approaches for enhancing NP applications. In addition to crosslinking, various polymers have been commonly used to enhance both the antibacterial properties and applications of chitosan NPs. Various types of polymers, such as polyphenols [52] and polysaccharides [112], have been combined with chitosan to enhance several physicochemical properties, including antibacterial activity.

Polyphenols are plant-based biomolecules that have attracted significant interest over the past few years owing to their significant potential benefits to human health, including antioxidant, antibacterial, anti-inflammatory, and antitumor activities [113]. This is why chitosan-polyphenol conjugations are suitable combinations in chitosan-based antibacterial applications since they both feature significant antibacterial characteristics. There is notable research that demonstrates the polyphenol-conjugated chitosan NPs’ antibacterial activity and their delivery applications, including diverse polyphenols [114,115].

One study used chitosan-based NPs as carriers to simultaneously deliver catechin and quercetin [116]. The dual-loaded NPs exhibited a slight increase in particle size, from 171.0 ± 2.7 nm to 190.7 ± 2.8 nm, and a reduction in zeta potential, from 32.57 ± 1.15 mV to 27.39 ± 1.71 mV. The particles were further modified with genipin, which adjusted the physicochemical properties to levels similar to those of the unloaded NPs. An improvement in antibacterial activity was observed through the reduction in MIC values. While individual administration of both polyphenols and chitosan MIC values between 19.53–156.25 μg/mL, dual-loaded particles significantly reduced the MIC value to 4.88–9.76 μg/mL against three different bacterial strains.

Polyphenol-conjugated chitosan molecules have also been utilized in various other applications. A well-characterized application involves using gallic acid-chitosan conjugates to modify NPs, particularly silver NPs, thereby enhancing their overall antibacterial activity [117,118]. In agricultural applications, various polyphenols can be conjugated with chitosan to produce films for food packaging as well. Wang *et al.* compared the antibacterial activity of two types of polyphenols, gallic acid, and caffeic acid, conjugated to chitosan films [119]. The study revealed that while polyphenol conjugation did not significantly enhance the antibacterial activity of certain strains, caffeic acid conjugation achieved a bacteriostasis rate of up to 95.24 ± 1.22%, whereas other groups ranged between 70.95 ± 3.20% and 87.88 ± 0.61% in certain types.

Polysaccharides are another common type of polymer that are widely used with chitosans. When recent applications are considered, alginate is one of the most widely used polysaccharides, along with chitosan. Chitosan-alginate-based NPs have been used in many applications, such as drug delivery systems, antimicrobial treatments, vaccine adjuvants, and agricultural applications [120].

Drug delivery systems utilizing alginate-chitosan NPs have demonstrated notable enhancements in their antibacterial activities. Almeleebia *et al.* conducted the co-delivery of naringin and ciprofloxacin using a chitosan-alginate NP composite [121]. Characterization of the NPs revealed that an increase in particle size from 50 ± 2.6 nm to 85 ± 8.5 nm significantly enhanced both encapsulation efficiency and drug-loading capacity. Larger particle sizes, such as 309 ± 14.6 nm, slightly reduced both encapsulation efficiency and drug-loading capacity. The antibacterial assay showed that the individually administered antibiotic exhibited an MIC value between 55 ± 4.4 and 67 ± 5 μg/mL, with a ZOI ranging from 23.56 ± 2.4 to 21.67 ± 4.67 mm. The addition of polyphenol reduced the MIC to 39 ± 6 μg/mL and increased the ZOI to 33 ± 4 mm. Delivery initiated with alginate-chitosan NPs demonstrated the lowest MIC value of 9.4 ± 1.6 μg/mL and the highest ZOI of 57.8 ± 6.2 mm. Additional studies, including alginate in chitosan-based NPs, are presented in the next Table.

Cellulose is another widely used polysaccharide incorporated into various chitosan-based nanomaterials, including NPs, hydrogels, scaffolds, and membranes [122]. Ju *et al.* demonstrated enhanced antibacterial activity through the incorporation of cellulose composite films with chitosan NPs [123]. The ZOI values indicated that cellulose composite films alone did not exhibit any antibacterial activity, whereas the addition of chitosan to the films generated antibacterial activity, with a maximum ZOI of 7.73  ±  0.20 mm at a chitosan concentration of 2.5% (*w*/*v*). In contrast, the incorporation of chitosan NPs significantly increased the ZOI value to 10.00  ±  0 mm at the highest concentration. A notable increase was observed in *S. aureus*, where chitosan NPs increased the ZOI by 4-fold. The addition of chitosan NPs further enhanced other features, particularly the UV barrier properties, highlighting their potential in food packaging applications.

Various types of proteins, particularly antimicrobial proteins, are also used with chitosan-based NPs. For example, lactoferrin (Lf) is a common glycoprotein that is widely used in NP applications. The main reason is that Lf is not only a promising antimicrobial protein with well-established multi-mechanisms [124], but has also recently been used as an NP itself for various applications [125]. This is why there are some studies that utilize the common features of both chitosan and Lf in various applications.

Duarte *et al.* synthesized Lf-chitosan NPs crosslinked with TPP and demonstrated their antibacterial activity and food preservation potential [126]. The characterization of NPs revealed that the zeta potential significantly increased to +39.30 ± 0.79 mV with minimal Lf concentration (0.035% *w*/*v*), minimal TPP concentration (0.010% *w*/*v*), and the highest chitosan concentration (0.055% *w*/*v*). In contrast, the zeta potential decreased to − 2.05 ± 0.06 mV with the highest Lf and TPP concentrations and the lowest chitosan concentration. The antibacterial activity test demonstrated that the highest zeta potential corresponded to the lowest MIC of 0.0370 mg/mL, which was approximately threefold lower than that of treatment with chitosan alone. Duarte *et al.* conducted a similar study, showing the antimicrobial activity of lactoferrin-chitosan-gellan NPs, highlighting their potential in food preservation [127].

Polymer conjugation and delivery with chitosan NPs have significant potential for antibacterial applications. Depending on the type of polymer, its surface chemistry and functional properties can be greatly altered. These alterations cannot only enhance their interaction with bacteria but also provide stability, enhanced release, and efficiency in many antibacterial-based applications.

### 4.2. Effect of Physicochemical Property and Concentration of Chitosan NPs on Nanocomplex-Based Antibacterial Applications

Various types of nanocomplexes, including hybrid NPs, NP-integrated hydrogels, and nanocomposites, have been designed to enhance antibacterial activity [128,129,130]. Chitosan is among the most preferred agents incorporated into these nanocomplexes for antibacterial applications, functioning both as a biomolecule and in the NP form. In these applications, both the concentration and physicochemical properties of chitosan NPs influence the activity of the complexes.

For instance, a study showed a change in the physicochemical properties of gold-chitosan hybrid NPs with respect to antimicrobial activity [131]. Chitosan concentration (up to 1000 μg/mL) was used as a variable to observe changes in the properties of the hybrid NP. With increased chitosan concentration, the following properties were observed: an increase in zeta potential from +25.1 to + 53.1 mV, a reduction in size from 34.7 ± 7.6 to 16.9 ± 3.9 nm (as measured by TEM), a proportional increase in the intensity of absorption bands, reduced aggregation, and increased thermal stability. Notably, the particle shape remained constant and spherical. As expected, these changes led to an enhancement in the antibacterial activity of the hybrid NPs. NPs synthesized with the highest chitosan concentration demonstrated the lowest MIC values against *S. aureus* (31.2 to 15.6 μg/mL) and *P. aeruginosa* (125 to 31.2 μg/mL). Additionally, a reduction in the MIC value was observed for *C. albicans* (250 to 62.5 μg/mL), demonstrating enhanced antifungal activity of the NP. It was emphasized that both size reduction and increased charge density (positive charge) contributed to the enhanced antibacterial activity of the particles.

Ahmet *et al.* developed TPP crosslinked chitosan NP hydrogels for testing the antibacterial efficiency of the structure [132]. The antibacterial activity was compared among crosslinked and modified chitosan NP-containing hydrogels, chitosan-only hydrogels, and sole administration of modified chitosan NPs and chitosan. Slight morphological differences were observed: chitosan hydrogels had a smooth surface, while crosslinked chitosan NP-included hydrogels exhibited a rougher and more irregular surface. The nanogels exhibited spherical and regular shapes, with slight size variations attributed to TPP crosslinking. The modified TPP-chitosan NP hydrogels demonstrated the highest thermal stability during the experiments. The *in vitro* antibacterial experiments were conducted on eight bacterial strains, including four Gram-negative and four Gram-positive. Bacterial inhibition was assessed using ZOI measurements, and all hydrogels exhibited slightly higher activity against Gram-positive bacteria. Chitosan modification increased the average ZOI values from 12 to 16 mm to 14.5–18 mm. Additionally, the incorporation of chitosan NPs with TPP crosslinking further increased these values from 9 to 17 mm to 16.5–20.5 mm. In conclusion, hydrogels containing crosslinked chitosan NPs exhibited the strongest antibacterial activity against Gram-positive bacteria, with the lowest MIC (19.5–31.2 μg/mL) and minimum bactericidal concentration (MBC) (38–58.5 μg/mL) values showing a 2- to 3-fold reduction compared to other formulations. Although the values were less significant for Gram-negative bacteria, the same formulation demonstrated the highest antibacterial activity.

Another study demonstrated changes in the physicochemical properties of starch nanocomposites based on the incorporation of chitosan NPs at various concentrations [133]. The study incorporated chitosan NPs at four different concentrations (1%, 2%, 3%, and 4%) into starch nanocomposites to enhance antibacterial activity for food packaging applications. As the concentration of chitosan NPs increased, the following changes in the properties of the nanocomposite were observed: a notable reduction in water absorption, water vapor transmission rate, and permeability (approximately 20% compared to non-chitosan-incorporated composites); a significant increase in Young’s modulus and tensile strength of up to nearly 3-fold; and substantial improvements in the overall mechanical properties. A highly dense morphology and rich nanofiller content were also observed. In terms of antibacterial activity, as expected, while the unloaded nanocomposite did not exhibit any antibacterial activity, the incorporation of chitosan NPs demonstrated significant activity against both Gram-positive and Gram-negative bacteria. The reduction in Gram-negative bacteria reached 81.77%, while Gram-positive bacteria showed a 100% reduction, demonstrating superior activity against Gram-positive bacteria.

Chitosan NPs can be utilized in antibacterial applications not only as an antimicrobial agent within various types of nanocomplexes but also by being synthesized with other types of NPs to form hybrids that enhance their applications. In these approaches, both the physicochemical properties of the chitosan NPs and the nanocomplexes they are incorporated into are significantly affected. Moreover, the conditions during their synthesis and the concentration incorporated during nanocomplex development further affect the efficiency of their application. In conclusion, variations in the antibacterial activity of chitosan NPs should also be considered in nanocomplex-based applications, taking into account their physicochemical properties.

## 5. Antibacterial Applications of Chitosan Nanoparticles

Chitosan is a promising and reliable biomaterial with significant antimicrobial potential, which has led to its use in diverse applications across various fields. Their high biocompatibility, reliable biodegradability, and strong antibacterial activity have enabled their use in various fields, including targeted antibacterial drug delivery, agriculture, environmental management, wound care, and dentistry. In this section, we have discussed the mentioned areas where both chitosan and chitosan NPs demonstrate their significant antibacterial activity and biocompatibility. In addition, we have briefly discussed the chitosan NP-based structures, primarily nanocomposites, in most of these applications.

Chitosan NPs have been used in various types of structures in biomedical applications, such as nanocomposites, complexes, and other types of NPs [134]. Table 3 summarizes some of the key studies from recent years (2020–2024), highlighting the continuous development of chitosan NPs applications and their impactful contributions to antibacterial research.

### 5.1. Antibacterial Applications of Chitosan and Chitosan NPs with Drug Delivery Systems

Chitosan NPs are recognized for their exceptional ability to transport various antibacterial agents while simultaneously exhibiting their well-known antibacterial activity. They can enable controlled drug release, be modified for targeted delivery strategies, cross biological barriers (such as the blood-brain barrier), and enhance the solubility, stability, and bioavailability of encapsulated drugs [154]. Moreover, numerous drug delivery systems have utilized chitosan-based films, nanocomposites, and various nanostructures, highlighting their usability and significance in the field [155] showing their significance in the area. As a result, a wide range of studies have employed chitosan NPs in delivery applications, including cancer therapy, gene delivery, vaccine delivery, and ocular drug delivery [156].

As highlighted in the previous section, physicochemical properties are heavily influenced during the development of drug delivery systems. Under certain conditions, antibacterial-based drug delivery strategies can enhance the overall activity of chitosan NPs and the antibacterial agents that they carry.

Gláucia-Silva *et al.* demonstrated the enhanced antibacterial activity of crosslinked chitosan NPs carrying *Tityus stigmurus* venom [157]. The synthesized spherical chitosan NPs exhibited a zeta potential of +23.20 ± 1.47 mV and a size of 134.40 ± 0.75 nm. After drug loading at 1%, the size decreased to 106.03 ± 1.94 nm, and the zeta potential increased to +26.96 ± 0.58, with an encapsulation efficiency of 78.67%. In addition, drug loading at 0.5% exhibited undesirable drug delivery characteristics, with a notable increase in particle size to 176.16 ± 1.45 nm, despite slightly higher zeta potential and encapsulation efficiency values (+ 28.63 ± 0.58 mV and 81.36%, respectively). The effect of size differences in the drug delivery system was reflected in the antibacterial efficiency. Against *S. aureus*, small-sized chitosan NPs exhibited 2-fold higher antibacterial activity, with a MIC value of 44.6 µg/mL, compared to 89.2 µg/mL for larger-sized particles. Interestingly, unloaded chitosan NPs exhibited higher antibacterial activity against *E. coli* than both types of drug-loaded particles. This study also emphasized that antifungal activity was enhanced by small-sized drug-loaded particles.

Another study showed enhanced properties of both chitosan NPs and *Eucommia ulmoides* seed essential oil, including antibacterial activity [158]. The encapsulated chitosan NPs showed an increase in encapsulation efficiency from 36.95 ± 1.62% to 67.80 ± 1.42% at a 1:0.75 drug concentration ratio. However, at higher drug concentration ratios (1:1 and 1:2.5), a slight reduction in encapsulation efficiency to 59.31 ± 1.85% was observed. Despite the reduction, the drug-loading percentage increased proportionally, reaching 7.50 ± 0.23%. As the drug loading increased, the zeta potential decreased to 17.4 ± 0.6 mV, while the particle size increased to 276.0 ± 16.6 nm. Despite their increased size and reduced zeta potential, antibacterial studies have revealed that drug-loaded chitosan NPs exhibit greater activity than both unloaded particles and the sole administration of the drug. In tests against three bacterial strains, chitosan and the sole drug treatment showed approximate ZOI of 2.5 cm and 4 cm, respectively, whereas drug-loaded particles exhibited a ZOI of 5 cm, outperforming both. Additionally, drug-loaded particles demonstrated superior biofilm prevention activity compared to the sole administration of the compounds. Furthermore, the antibacterial effects of these compounds were compared based on their administered concentrations. While chitosan alone induced cell lysis and destruction at a high concentration of 1280 μg/mL, drug-loaded particles caused initial morphological changes at 80 μg/mL and signs of destruction at 320 μg/mL. At the same concentration of 1280 μg/mL, the drug-loaded particles nearly achieved complete destruction of bacterial cells.

Antibacterial drug-loaded delivery strategies utilizing chitosan NPs can significantly enhance the effectiveness of antibacterial therapies. As highlighted in the referenced studies, drug-loading applications can outperform both the sole administration of chitosan and drugs alone. However, it is important to note that drug loading significantly alters the physicochemical properties of the particles, which in turn affects their activity and stability. Given the alterations in size and zeta potential, the resulting changes in antibacterial activity may not be significant for certain bacterial strains. Nonetheless, chitosan NPs offer significant advantages for drugs because of their poor bioavailability, solubility, and stability [159]. Additionally, they can facilitate controlled drug release and targeted delivery through surface modification. Therefore, with precise control over their properties, chitosan-based NPs can provide significant advantages for developing novel drug delivery strategies in the future.

In addition to the direct utilization of chitosan NPs in drug delivery systems, a variety of studies have used chitosan NPs in various nanocomposites to enhance delivery strategies. The addition of chitosan to nanocomposites not only enhances the expected antibacterial activity but also increases biocompatibility, thermal stability, mechanical properties, and bioactivity, leading to their involvement at high concentrations [9].

Sanmugam *et al.* demonstrated enhanced antibacterial and drug delivery capabilities of chitosan-based reduced graphene oxide-CeO_2_ nanocomposites [160]. The synthesized nanocomposite exhibited the following features: increased thermal resistance, crystalline and rough structure, faster and sustained drug release, and higher optical transparency compared to chitosan and chitosan-based reduced graphene oxide. In terms of antibacterial activity, chitosan demonstrated a ZOI diameter of 12 ± 0.18 mm for both *E. coli* and *S. aureus*. The addition of reduced graphene oxide doubled this value (25 ± 10.31 mm), while the synthesized nanocomposite increased it nearly fourfold (42 ± 0.84 mm). The researchers emphasized the higher antibacterial activity against Gram-positive bacteria, attributing it to interactions initiated with the peptidoglycan layers of the bacterial cell walls. Additionally, researchers have highlighted the typical electrostatic interaction between the positively charged nanocomposite and negatively charged bacterial residues.

A similar study investigated the pH-sensitive behavior of polysaccharide–chitosan NP nanocomposites in terms of their drug delivery and antibacterial capability [161]. The synthesized composite NPs had an approximate size of 153 nm and spherical morphology at pH 7.4. The composite failed to maintain its structure and exhibited significant aggregation at pH 10, while forming large, irregular, and loosely shaped particles at pH 4.0. Nanocomposites at a pH of 7.4 exhibited the lowest zeta potential, measured at −20.31 ± 1.6 mV. Antibacterial activity tests revealed that the synthesized nanocomposite particles were most effective against Gram-positive *Staphylococcus epidermidis* (*S. epidermidis*), destroying 45% of the bacteria. Against Gram-negative *E. coli*, the nanocomposites achieved a bacterial destruction rate of 30%, making them the second-most effective agents in the experiment. Pure chitosan showed the highest antibacterial activity, destroying nearly 60% of *S. epidermidis*. However, it was not significantly effective against *E. coli*, with a destruction rate of approximately 17%. The drug delivery capacity of the composite particles was tested using three different types of drugs at acidic, basic, and natural pH levels. In an acidic environment, the composite showed the fastest drug release, followed by that in the natural environment. The *in vitro* experimentation and release kinetics highlighted the significant drug carrier capability of the composite particles.

Chitosan is typically utilized in nanocomposites in its natural form rather than as an NP structure. Additionally, studies often examine the antibacterial and drug delivery capabilities of chitosan separately, focusing more on characterization than application. However, the current research highlights the significant potential of chitosan composites in both drug delivery and antibacterial applications, with the possibility of integrating these functionalities in future studies. The most important point to highlight is the variability in composite properties and their differing effectiveness against Gram-negative and Gram-positive bacteria. Based on the antibacterial activity of chitosan NPs, developing and optimizing chitosan-based composites for specific pH levels and bacterial strains could be more effective than traditional chitosan NP-based applications.

### 5.2. Antibacterial Application of Chitosan and Chitosan NPs in Agriculture

Chitosan and chitosan NPs have been widely employed by researchers in the field of agriculture owing to their immense antibacterial activity. Primarily because of their antibacterial activity, they are being incorporated into food packaging and food preservation systems to enhance the shelf life of fruits and vegetables [162].

For example, Sree *et al.* developed edible chitosan coatings with different concentrations (0.5%, 1%, 2%, and 2.5%) to reduce post-harvest loss of tomato fruit by delaying the ripening process [163]. After 30 days of application at 30 ± 3 °C, the coated tomatoes remained less decayed, firmer, and higher in titratable acidity. In contrast, the control group demonstrated rapid deterioration after only 20 days of storage, supported by an increase in shrinkage from 0% to 30.57%. However, the chitosan-coated samples demonstrated a maximum shrinkage of 25.98%, even at the lowest concentration of 0.5%.

Similarly, researchers utilized chitosan in the form of NP to develop bioplastic packaging materials [25]. Modifying NPs with various molecules, including polyethylene glycol methyl ether methacrylate (PEGMA), stearyl methacrylate (SMA), and deoxycholic acid (DC), they obtained three different chitosan NP derivatives within a size range of 25 to 60 nm. Later, these derivatives were incorporated into polylactic acid (PLA) films, and their bactericidal activity was evaluated. The results revealed immense antibacterial activity against *S. aureus* by all the modified NPs, as evidenced by an average inhibition rate of more than 98%. In contrast, the antibacterial activity of the films containing chitosan-PEGMA and chitosan-DC NPs was found to be 0.20 CFU/mL (36.84%) and 0.69 CFU/mL (79.29%), respectively, when tested against *E. coli.* However, chitosan-SMA NP-incorporated films remained the most potent, with an antibacterial activity of 1.33 CFU/mL (95.33%). In addition, the application of chitosan-SMA NP-containing films on bread slices showed inhibition of the growth of microorganisms as well as reduced levels of mold expansion following five days of storage.

Hosseini *et al.* synthesized cinnamaldehyde-loaded chitosan NPs (CCNPs) and integrated them into a ternary film matrix containing chitosan/poly(vinyl alcohol)/fish gelatin (CPF) [164]. CPF-CCNPs demonstrated enhanced antibacterial activity against both Gram-negative (*E. coli* and *Salmonella enteritidis*) and Gram-positive (*S. aureus* and *L. monocytogenes*) food-borne pathogens in comparison to the CPF alone. In addition, the application of packaging extended the shelf life of rainbow trout fillets from 8 to 12 days, as CPF-CCNPs effectively controlled bacterial growth. The films maintained the total viable count (TVC) at 6.29 log CFU/g and prevented it from exceeding the acceptable limit of 7 log CFU/g following 12 days of storage.

Apart from these studies incorporating chitosan NPs into food packaging materials, there is also an increasing number of research focusing on the formulation of chitosan NP-containing food protection solutions.

For example, Alarfaj *et al.* investigated the use of antibacterial chitosan NPs at different concentrations (10, 25, 50, 100, and 150 µg) for protection against the food-spoilage bacteria *Bacillus* sp. and *Pseudomonas* sp. [26]. The results revealed notable bactericidal activity of chitosan NPs, with more pronounced effects at elevated concentrations. In particular, an increase in the ZOIs was observed, from 14 to 18 mm for *Bacillus* sp. and from 12 to 19 mm for *Pseudomonas* sp., when the chitosan NP concentration was increased from 100 to 150 µg.

Similarly, Paomephan *et al.* developed a chitosan NP-incorporated vegetable wash disinfectant and focused on the effect of physical properties on the efficiency of the synthesized NPs [165]. In their study, they utilized NPs of three different sizes and two different molecular weights. Comparative experiments on *E. coli* revealed that smaller-sized chitosan NPs, either at low or high molecular weight, had superior antibacterial activity by leading to a 2 log reduction in the number of bacteria within 12 h. Further, chitosan NPs were formulated in 1% citric acid to enhance the overall antimicrobial activity and tested for their effectiveness. The results revealed a 3.38 log CFU/mL reduction in *E. coli* by the smallest NPs, while the largest NPs led to a 2.83 log CFU/mL reduction in the number of *S. typhimurium* within 15 min. Finally, when the solution is applied to fresh lettuce, it leads to more than 1 log reduction in the bacterial population, confirming the promising potential of the final product.

In addition, chitosan NPs are regarded as crucial materials to replace commercially used chemical pesticides owing to their non-toxic and biodegradable nature.

As an example, Sreelatha *et al.* synthesized thymol-loaded chitosan NPs (TCNPs) against the plant bacterial pathogen *Xanthomonas campestris pv. campestris.* (Xcc) [166]. *In vitro* antibacterial assays demonstrated significant inhibition of Xcc by TCNPs within the range of 100 to 600 μg/mL. In addition, the percentage inhibition of Xcc increased with increasing NP concentration, reaching nearly 80% at the concentration of 600 μg/mL. These findings collectively indicate the promising potential of chitosan NP, including nanopesticide formulations, to control the growth of plant pathogens.

Khairy *et al.* investigated the use of chitosan NPs against bacterial wilt in potato and tomato. Bacterial wilt is one of the most destructive diseases associated with *Solanum* spp. and is known to be caused by the soil-borne bacterium *Ralstonia solanacearum* (RS) [167]. Conducting experiments on three different RS isolates (RS1, RS3, and RS5), researchers recorded the largest inhibition zones at the highest NP concentration (200 μg/mL), as 2.59, 3.10, and 2.00 cm for RS1, RS3, and RS5, respectively. Additionally, *in vivo* spraying of the 200 μg/mL chitosan nanoformulation reduced disease incidence and severity in potato plants by 78.93% and 71.85%. When applied to tomato plants at the same concentrations, NPs led to reductions in disease incidence and severity of 81.64% and 77.63%.

Considering these results, chitosan NPs, either alone or combined with various biomolecules, can be regarded as promising nanomaterials in the field of agriculture. Being immense antibacterials, they can be incorporated into food packaging materials and food preservation solutions or used as natural alternatives to commonly used chemical pesticides. In addition to these applications, their employment also extends to various aspects, including plant growth regulation studies to increase crop yields [168,169,170]. Hence, further optimization and widespread use of chitosan NPs will not only advance antibacterial studies, but also hold great potential to provide novel strategies in the industrial area.

### 5.3. Chitosan NPs in Water Disinfection

Chitosan NPs are of great importance in industrial areas, especially in wastewater treatment, owing to their desirable characteristics, such as high adsorption potential and ability to chelate metallic cations effectively [171]. In this manner, their employment for the removal of heavy metals, including lead, mercury, copper, chromium, and dyes from aqueous solutions has significantly increased [172,173,174]. However, the use of chitosan NPs in the industrial field is not limited to these applications but also extends to the purification of pathogenic bacteria from water.

For example, Denisova *et al.* investigated the disinfection capability of chitosan NPs in drinking water [175]. In their study, NPs of three different molecular weights (low, medium, and high) at different concentrations (0.25, 0.5, and 2%) were synthesized. Antibacterial tests on tap water containing approximately 5 × 10^5^ CFU/mL of bacteria revealed a 4.0 ± 0.06 reduction and 1.5 ± 0.11 log inactivation in cultivable and metabolically active *E. coli*, respectively, by 0.25% medium molecular weight NPs following 6 h of exposure. In addition, when contact time was increased up to 24 h, enhanced bactericidal effects with 5.9 ± 0.09 log reduction for cultivable and 4.9 ± 1.1 log reduction for metabolically active bacteria were observed.

Garcia Peña *et al.* developed cork matrices embedded with hybrid chitosan-silver NPs to reduce microbial contamination in drinking water [176]. *In vitro* assays on samples containing approximately 10^7^ CFU/mL of *E. coli* revealed 4 and 5 log reductions in bacterial counts after two 15 min disinfection cycles. In addition, complete removal of bacteria was achieved when the water residence time was increased from 15 min to 8 h.

From another perspective, various studies have used chitosan as a platform in nanocomposite form. Motshekga *et al.* established novel antibacterial bentonite-chitosan nanocomposites by incorporating bentonite containing silver and ZnO NPs, either alone or combined, into the chitosan matrix [177]. *In vitro* tests on *E. coli* and *Enterococcus faecalis* contaminated water (at concentrations of 500, 5000, and 50,000 CFU/mL) demonstrated superior results when silver and ZnO NPs were used in combination, leading to a minimum removal efficiency of 78%. The same formulation also led to total inhibition of both bacterial counts at 500 CFU/mL within just 2 min of exposure. However, when NPs were administered individually, the ZnO NP-containing samples showed stronger results by achieving complete bacterial inactivation within 10 min, outperforming the formulations of silver NPs, which required 15 min. Moreover, researchers have demonstrated enhanced activity towards *E. coli*, which is attributed to the thinner cell wall of Gram-negative bacteria in comparison to their Gram-positive counterparts.

Another application where chitosan NPs’ water disinfection capability is exploited is the water injection method, which oil- and gas-producing companies widely use to purify microorganisms and biofilms from seawater. In this aspect, Rasool *et al.* demonstrated that ZnO-interlinked chitosan NPs are capable of inhibiting sulfate-reducing bacteria (SDR), regarded as one of the primary factors that affect water safety [178].

Considering these studies and the given applications of chitosan NPs and chitosan-containing nanosystems in wastewater treatment, their large-scale employment will not only promote a safer environment but also contribute to overcoming economic challenges in industrial areas. Therefore, it would be crucial to focus on the development of chitosan-incorporating nanosystems and promote their wider use in further research.

### 5.4. Chitosan Nanoparticles in Wound-Healing Applications

The use of nanotechnology in wound healing research is regarded as a promising approach for the development of novel therapeutic systems [179]. Recently, various studies in the current literature have highlighted the effectiveness of NP-incorporated wound dressings with superior antimicrobial capabilities [180,181,182].

In this aspect, bio-safe chitosan NPs come forward with their distinctive antibacterial activity combined with high drug-loading capability to enhance and accelerate the wound care process [31].

For example, Fahimirad *et al.* developed poly(ε-caprolactone)/chitosan/curcumin nanofibers electrospray with curcumin-loaded chitosan NPs (CURCSNPs) [183]. Following the incorporation of CURCSNPs into the nanofibers resulted in enhanced antibacterial activity against MRSA and *E. coli,* with 99.3% and 98.9% growth inhibition rates after 48 h, respectively. Further *in vivo* assays on the mouse model revealed 96.4% and 98.5% healing percentages in 1.5 × 10^8^ CFU/mL MRSA-infected and non-infected wounds following 15 days of treatment. In addition, CURCSNP-containing nanofibers demonstrated the highest bactericidal efficiency compared to their counterparts, leading to complete inactivation of bacterial growth at the end of day 10. The same formulation also promoted quicker healing, supported by better epithelialization, improved well-organized granulation tissue, and reduced lymphocyte and neutrophil infiltration.

In another study, Thao *et al.* investigated the wound-healing activity of novel *N*-succinyl (*N*-SuC) chitosan NP films, a water-soluble derivative of chitosan that is known to play a major role in wound-healing acceleration due to its desirable properties [184]. *In vitro* tests on both Gram-positive *S. aureus* and Gram-negative *E. coli* revealed potent antibacterial activity of *N*-SuC NP films, with MIC values of 8 mg/mL and 6 mg/mL, respectively. In addition, the wound-healing capability of the NP-containing film was found to be superior to that of *N*-SuC in its natural form, as indicated by the percentage of open wound area rates after 36 h, 15.25% for *N-*SuC and 5.25% for *N*-SuC NP films. Similarly, *in vivo* administration of *N*-SuC NP-containing films to Wistar rats (with wound defects of 8 mm in diameter) accelerated the healing process by leading to 84.21% wound closure, compared to the *N*-SuC film with 72.48%, following 9 days of treatment.

Alternatively, chitosan NPs have the potential to be combined with other antibacterials to strengthen bactericidal efficiency and improve overall wound-healing effects [185]. Focusing on this, researchers have synthesized chitosan NPs loaded with recombinant LL37 antimicrobial peptides (CSLL37NPs) [186]. Further experiments on *E. coli* and MRSA revealed superior activity of CSLL37NPs in comparison to free chitosan NPs and LL37, as evidenced by lower MIC values. Specifically, exposure to CSLL37NPs at concentrations of 32 µg/mL and 16 µg/mL, both representing twice the MIC value, led to three log reductions in the number of viable cells within 60 min, which then resulted in complete bacterial inhibition after 80 and 100 min of exposure against *E. coli* and MRSA, respectively. On the other hand, LL37 alone achieved the same results after 120 min, while chitosan NPs alone caused a 6 log reduction in bacterial count but were unable to reach complete inhibition in this period of time.

Given the broad range of applications of antibacterial chitosan NPs in wound-healing studies, it is possible to develop biodegradable, non-toxic, and non-allergenic formulations that would facilitate an accelerated way of healing while promoting tissue regeneration and reducing the risk of infections.

### 5.5. Chitosan Nanoparticles in Dental Applications

NPs have diverse applications in dentistry, particularly in the treatment of caries and periodontal diseases, as they are incorporated into dental implants and resin nanocomposites [187]. Chitosan is a commonly preferred molecule for many dental applications. It is used in oral products such as toothpaste and mouthwashes, as an additive in prosthodontics and endodontic therapies, among other applications [38]. Chitosan NPs are used in dentistry to promote mineralization, contribute to tissue engineering structures, and, most importantly, prevent bacterial growth [188]. The antibacterial activity of chitosan NPs is a key feature driving their application in dentistry, and is implemented through various strategies.

One approach to employing chitosan in dentistry involves utilizing its drug delivery capability for the treatment of caries. Zhu *et al.* demonstrated the anti-caries effect of chitosan-based nanogels with dual drug encapsulation [189]. The drug-loaded chitosan nanogels had an average size of 260.2 nm and a zeta potential of −16.3 ± 3.97 mV. The characterized hydrogel was tested for its potential biofilm effect and antibacterial activity against *S. aureus*. Colony counts following co-treatment with the nanogels showed a rapid and significant reduction within five minutes to 3.10–3.45 CFU/mL, compared to 14.45–15.70 CFU/mL with double-distilled water treatment. The colony count was further reduced to 0.45–0.76 CFU/mL after 24 h. Furthermore, *Streptococcus mutans* biofilms were grown on teeth for two days and then treated with hydrogels to evaluate their anti-biofilm capability. The nanogels significantly reduced biofilm levels to 55.7%, compared to 89.9% in the double-distilled water-treated groups. Finally, the nanogels exhibited the lowest surface hardness loss of 29.2%, highlighting their potential in anti-demineralization applications.

Another study utilized chitosan-silver NPs to modify glass ionomer cement to enhance its antibacterial activity [190]. The antibacterial activities of chitosan NPs and silver NPs were tested separately using the disc diffusion method. Chitosan NPs exhibited an average inhibition zone of 9.87 mm, outperforming the 0.2% silver NP solution (8.52 mm), while the 0.5% silver NP solution showed the highest activity with a 13.87 mm inhibition zone. The primary experiment involved a biofilm test using various concentrations of NPs incorporated into glass ionomer cement. The results showed that chitosan NPs (10%) and silver NPs (0.5%) individually reduced biofilm levels to an average of 172.5 CFU/mL and 168.5 CFU/mL, respectively. Although the difference was not statistically significant, the combined modification with both types of NPs achieved the greatest reduction, lowering the biofilm levels to 165 CFU/mL. These findings suggest that chitosan NPs not only hold significant potential for use in dental fillings, but can also complement other types of NPs to enhance treatment efficacy.

To give one last example, Pourhajibagher *et al.* applied a combination of photothermal therapy (PTT) and photo-sonodynamic therapy (PSDT) using chitosan NPs loaded with the photosensitizer indocyanine green to target periopathogenic bacterial biofilms on dental implants [191]. The study synthesized small, spherical NPs approximately 15 nm in size with a zeta potential of −3.6 mV. This study evaluated biofilm reduction and CFU counts across various treatment groups. PTT is a strong strategy that can possess high efficiency in antibacterial applications, which was also observed in this study with 54.4% biofilm reduction to a mean value of 4.43 ± 0.12 CFU/mL. Incorporating chitosan NPs into the PTT group increased the inhibition ratio to 67.2%, reducing the mean CFU count to 3.18 ± 0.13 CFU/mL. PSDT alone showed similar efficacy to PTT, achieving a 57.9% biofilm reduction with a mean CFU count of 4.09 ± 0.10 CFU/mL. Adding chitosan NPs to PSDT increased biofilm reduction to 68.4%, highlighting the comparable efficacy of the therapies and the contribution of chitosan to the treatments. Notably, the combined therapy achieved a 73.2% biofilm reduction, which increased to 90.5% with the addition of chitosan NPs, lowering the CFU count to 0.92 ± 0.14 CFU/mL.

Chitosan NPs have extensive applications in dentistry, primarily due to their drug delivery capabilities and antibacterial properties. Similar to its role in wound-healing applications, chitosan serves as a valuable biomaterial for coating dental drug carriers and nanostructures [192]. Given the importance of antimicrobial NPs in dental applications, the strong antibacterial potential of chitosan NPs requires greater attention in future research.

## 6. Future Perspective

As a material, chitosan possesses wide-scale applications in important fields like the food industry, agriculture, cosmetics, water treatment, and biomedical areas [193]. Considering the molecular advantages that nanomaterials possess, using chitosan in these materials certainly enhances the efficiency of these structures, as highlighted in most of the sections in the review. NPs are among the foremost nanomaterials that are predominantly used in nanotechnology research. With the increased focus on NP research in the current literature, chitosan will be of great importance as one of the leading materials because of its non-toxic and biodegradable nature.

Chitosan is already an ongoing bioactive polymer that is widely used in various nanomaterials, including hydrogels, nanocomposites, and NPs, as a supportive material. Considering the emerging need for green-synthesized and eco-friendly nanomaterials in many areas, chitosan is an ideal material to support the development of these areas. In addition, chitosan plays a significant role in the green synthesis of metal NPs for biomedical applications since it possesses an important role as a reducing agent, along with its influence on the stability, shape, and size of the particles [194]. This is why further research should focus on the integration of chitosan with eco-friendly polymers, metal NPs, or bioactive molecules in hybrid systems and functionalized nanomaterials.

Moreover, chitosan NPs are considered efficient antibacterial agents, as much research has shown their bactericidal activity in sole administration, mostly by their association with bacterial cell walls or membranes. As we have discussed in previous sections, chitosan NPs demonstrate bactericidal activity through various mechanisms, including electrostatic interaction, chelation of metal ions, and alteration of transport pathways of bacteria. However, electrostatic interaction is the most predominantly mentioned among these mechanisms, where positively charged amino groups of chitosan NPs bind to the negatively charged cell membrane. In light of this, there is ongoing research on the development of chitosan NP-based nanomaterials, including nanofilms, nanotubes, and nanoformulations, to exploit and extend the potent antibacterial activity of chitosan NPs into diverse fields. A comprehensive understanding of these mechanisms is considered crucial for the further design and development of such nanomaterials.

**Figure 5:**
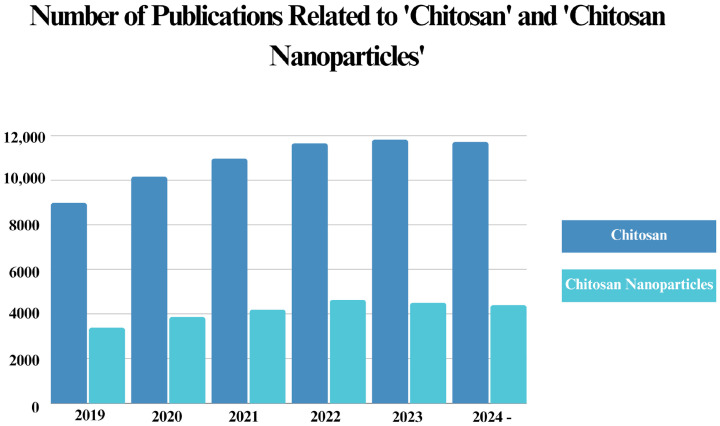
Number of publications on “Chitosan” and “Chitosan Nanoparticles” published in the Web of Science Core Collection over the last five years. The obtained publication numbers were searched with a “Topic” filter, representing titles, abstracts, and keywords that included the highlighted words [195].

In addition to their incorporation into other types of nanomaterials, chitosan NP themselves require sufficient research effort, as there is a large research background showing their efficiency as drug carriers and antimicrobial agents. Thankfully, chitosan has gained significant interest due to its advantages and biocompatibility. In addition, chitosan NPs have a similar interest with a notable increase, as shown in Figure 5.

Based on the data acquired from the Web of Science Core Collection, the annual number of publications related to “chitosan” has shown an increase, with nearly approaching 12,000 per year. In addition, approximately 40% of these publications consisted of “chitosan nanoparticles”, highlighting the significance of nanomaterials in current chitosan research.

Researchers have mainly focused on biomedical applications, including the development of delivery systems, wound-healing formulations, and bactericidal agents incorporated with chitosan NPs [15]. Nonetheless, the literature contains gaps that hinder the research on chitosan NP. From the studies we have cited and discussed, several critical factors are notably absent: a detailed explanation for the differences in antibacterial efficiency between Gram-positive and Gram-negative bacteria, the impact of chitosan’s physicochemical properties (specifically molecular weight) on antibacterial mechanisms, and *in vivo* studies elucidating both the mechanisms and toxicity conditions of chitosan-based nanomaterials [72]. Although chitosan NPs demonstrate well-established antibacterial activity, further investigation is still needed. In addition, the distribution of research efforts towards antibacterial applications requires improvement. While antibacterial-based drug delivery applications constitute the majority of chitosan-based research, other areas, such as wound healing and dental applications, have received insufficient attention. Chitosan has a significant potential to advance these fields and alleviate some of their major challenges. For instance, a key challenge in wound-healing applications is synthesizing hydrogels with a degeneration rate that matches their regeneration speed [196]. Given the biodegradable and significant antibacterial properties of chitosan, hydrogels incorporating chitosan are worth further investigation for this purpose. However, research efforts on wound-healing applications using chitosan-based nanomaterials remain insufficient compared to other antibacterial applications in which chitosan is utilized.

Moreover, the use of chitosan NPs in agriculture, as additives in food packaging and food preservation systems, and in industrial areas, particularly in water disinfection formulations, should not be ignored [28]. As food packaging materials should enhance the biodegradability of the package, support mechanical properties that ensure the quality and safety of the food, and confer a notable antibacterial activity for preventing contamination and food spoilage, chitosan is uniquely suitable for this purpose [197]. Similarly, chitosan NPs hold significant potential in this area, especially when included in various nanocomposites. However, it is worth mentioning that physicochemical properties have a great impact on these applications, as they comprise most of the current drawbacks in the field [28].

Given the immense effectiveness of chitosan NPs in diverse fields, it will be possible to conduct large-scale experiments for commercial use in the near future. As an example, researchers are developing chitosan NP-containing nanofertilizers and nanopesticides to increase crop yields and enhance the durability of plants against pests and diseases [198,199]. In addition, chitosan NPs can be employed to remove pollutants and heavy metal ions from wastewater, which is one of the main issues associated with industrial discharges [172]. The main reason for this is the significant antimicrobial activity of chitosan, which allows it to bind and neutralize contaminants, as well as its high adsorption capacity for heavy metal ions due to the presence of amino and hydroxyl groups in its structure.

Regarding antibacterial efficiency, another crucial aspect to highlight is the physicochemical properties of chitosan NPs and chitosan-based nanomaterials, as these characteristics significantly affect their functionality and application potential. Thus far, we have discussed and emphasized the impact of physicochemical properties, such as zeta potential and size, on the antibacterial activity of both chitosan NPs and chitosan-based nanomaterials. In addition to eco-friendly approaches, optimizing the synthesis methods and refining these properties are essential for improving the large-scale application of these materials. The synthesis method and conditions significantly influence the primary attributes of NPs, specifically their size, shape, and surface chemistry [200]. As these characteristics significantly affect the antibacterial mechanisms, properties determined through synthesis methods can enhance or reduce the efficiency of chitosan NPs. Factors such as particle shape and environmental conditions, including pH and temperature, can significantly influence the antibacterial activity [201]. Moreover, the characteristics of chitosan itself, specifically molecular weight and DD, play a significant role in its antibacterial efficiency [202]. The effects of some of these factors on antibacterial mechanisms have been well established. For instance, increased DD enhances antibacterial activity by increasing the charge density of chitosan NPs, thereby strengthening electrostatic interactions [203]. A similar pattern is observed when pH is varied during antibacterial studies. Since chitosan’s amino group exhibits a positive charge at pH levels lower than 6.3 [204], the efficiency of electrostatic interactions is greatly influenced. However, there are other factors that need details to reveal their influence on the antibacterial mechanism. Molecular weight is a notable example, as variations in molecular weight and concentration yield differing antibacterial results without clear justification regarding their mechanisms [205]. Finally, although many of these characteristics enhance the mechanisms, certain factors must be carefully monitored during the synthesis procedures. One major feature is stability, which significantly influences the aggregation of chitosan NPs. These findings show the need for optimization and additional comparative studies focusing on antibacterial mechanisms

To advance NP-based product development, including chitosan-containing nanomaterials, their physicochemical properties must be precisely characterized, as these properties strongly influence their condition within the body [206]. Further research and reviews addressing this issue can support the development of clinical trials by resolving the many concerns associated with general nanomaterials.

## 7. Conclusions

Chitosan NPs have achieved a significant position in several sectors and scientific domains since their first characterization about two decades ago. A variety of preparation methods have emerged, including environmentally friendly techniques that exclude potentially hazardous or toxic substances, such as spray drying and supercritical CO_2_-assisted procedures.

The potential of chitosan NPs as antibacterial agents has been extensively demonstrated in diverse fields, including agriculture, environmental management, antimicrobial drug delivery systems, and dental biomaterials. Due to their biocompatibility and high biodegradability, chitosan continues to be widely explored for many antibacterial applications. In addition, the compatibility of chitosan and chitosan NPs with various structures is a significant factor that advances their employment in antibacterial research. These structures can lead to the development of novel strategies and relieve the difficulties associated with antibacterial solutions. The use of chitosan NPs in agricultural practices and wastewater treatment offers a significant opportunity to replace commercially available chemicals with chitosan-based materials. Moreover, their significant drug delivery capability can help combat drug-resistant bacteria, thereby addressing one of the most pressing challenges in health sciences. However, the physicochemical properties of both chitosan NPs and chitosan-containing structures are crucial and require significant attention during synthesis and further application. Despite their significant potential, the optimization of chitosan NPs’ antibacterial efficacy under diverse environmental and physiological conditions remains insufficient to address the challenges of upscaled synthesis and application. Further research aimed at enhancing both the functionality and physicochemical properties of chitosan NP-based structures could significantly advance their practical applications, which is considered a critical focus in chitosan research.

## Figures and Tables

**Table 1:** Synthesis methods of chitosan NPs and their advantages and limitations.

Synthesis Method	Principle	Advantages	Limitations	References
Ionic gelation	The electrostatic interaction of a polyanion (such as TPP) with chitosan	Simple, mild, and eco-friendly	Limited particle size control; sensitive to ionic strength	[62]
Emulsion-Droplet Coalescence	NPs are formed in a water-oil system via solvent diffusion or evaporation.	Uniform particles suitable for hydrophobic drugs	Requires organic solvents; time-consuming	[44]
Spray Drying	Chitosan solution atomization and solvent evaporation	Produces dry, stable powders; scalable	High energy process; potential loss of bioactivity for sensitive molecules; large particle size	[50]
Self-Assembly	Chitosan molecules assemble spontaneously under some circumstances	No organic solvents; suitable for biomolecules	Sensitive to pH and ionic strength	[51]
Reverse Micellar Method	NPs generated in microemulsions of water and oil	Produces small, uniform particles	Complex process; use of organic solvents	[43]
Chemical Crosslinking	Crosslinked NPs are produced with substances like glutaraldehyde.	Produces stable NPs with tunable properties	Use of potentially toxic crosslinkers	[38]
Supercritical-CO_2_- assisted solubilization and atomization	Atomization	Solvent-free method; does not require additional separation process	Large particle size; time-consuming process.	[38]
Phase inversion precipitation	Precipitation	Suitable for large-scale production, simple and cost-effective	Requires organic solvent, which can be toxic, limited control for particle size and morphology	[45]
Ionic gelation with radical polymerization	Polymerization and crosslinking	Precise control for particle size and morphology, suitable for drug delivery applications	Complex synthesis procedure, high cost	[48]
Free Radical Grafting	Graft copolymerization.	Cost-efficient reaction reagents, lower toxicity, no requirement for heat, utilization for polysaccharides without amino group.	Low efficiency, limited specificity, possibility of the degradation of polysaccharides during the process.	[63]
Polyelectrolyte Complex Method	Electrostatic interaction between chitosan (positively charged) and a polyanion (e.g., TPP).	Simple, mild, eco-friendly, no organic solvents required.	Limited particle size control, sensitive to pH and ionic strength.	[64]
Electrospraying	High-voltage electrostatic force generates fine droplets containing chitosan solution. These droplets solidify to form NPs	Consistent particle size, enhanced encapsulation efficiency, appropriate for both hydrophilic and hydrophobic pharmaceuticals.	Requires high-voltage apparatus, constrained scalability, and solvent evaporation challenges.	[65]
Top-down	Acid hydrolysis and deacetylation	Scalable for industrial application, precise control for particle size and morphology	High energy consumption, including harsh reaction conditions	[60]

**Table 2:** Effects of the Physicochemical Properties of Chitosan and its NPs on the Antibacterial Activity.

Features of Applied Chitosan NPs	Physicochemical Properties	Enhanced Activity Results	References
Crosslinked chitosan NPs for antibacterial drug delivery	Average size of 478 ± 86 nm Zeta potential of −29.2 ± 1.1 mV	-High drug loading capacity. -Temperature-dependent antibacterial activity with high inhibition zones	[88]
Crosslinking of chitosan nanocomposite with silver-sulfur doped graphene quantum dots	Increased optical peak with crosslinking	-Enhanced antibacterial activity with higher crosslinking levels of quantum dots.	[89]
Antibacterial activity of nanoscaled crosslinked chitosan with citric membranes	Reduced tensile strength and increased elongation at break High oxygen barrier capability	-Significant enhancement in antibacterial activity.	[90]
Crosslinked chitosan included nanocomposite for improved antibacterial and mechanical properties	3.5-fold increase in compressive strength	-Enhanced antimicrobial activity with increased anti-biofilm activity.	[91]
Using capping agents on chitosan-gold hybrid NPs for enhancing antibacterial activity	Spherical morphology Increased zeta potential from −26.4 ± 6.3 to 31.0 ± 6.0 mV Increased particle size from 5.0 ± 4.0 to 34.1 ± 5.9 nm	-Enhanced antibacterial activity with reduced MIC values.	[92]
Enhanced delivery of antibacterial agents with chitosan NP thioliation	Average size of 136.26 ± 43.17 nm upon drug loading Spherical morphology	-High encapsulation efficiency. -Enhanced antibacterial drug delivery with reduced MIC values.	[93]
Effect of differently crosslinked chitosan NPs in antibacterial activity of zinc oxide (ZnO) NP-included nanocomposite	Except for elemental analysis, no notable changes were observed in the physicochemical properties of the nanocomposites.	-Significant antibacterial activity with reduced MIC values.	[94]

**Table 3:** Antibacterial applications of chitosan and chitosan NPs in recent years (2020–2024).

Research Material	Properties	Results	References
Peptide-capped chitosan-gold NPs	Size: ~227 nm Spherical Morphology Zeta potential: +42 mV	-Uncapped NPs decreased colony-forming unit (CFU) values to 136 ± 13 and further decreased with laser irradiation to 103 ± 6. -Capped particles significantly reduced the CFU values to 81 ± 3 and further decreased with laser irradiation 69 ± 4.	[135]
Chitosan NP-incorporated whey-based Poly (L-Lactic Acid) (PLLA) packaging films	Thickness: 70–80 μm	-Increased water vapor permeability and elongation at break. -Enhanced antibacterial effect by increased chitosan NP concentration. -Improved tensile strength and Young’s modulus.	[136]
Nickel oxide (NiO) NP-incorporated chitosan-based nanocomposite films	Thickness: 25–31 mm.	-Antibacterial activity against both Gram-positive and Gram-negative bacteria. -Photocatalytic activity evidenced by 72% methyl orange absorption following 270 min of exposure to UV radiation.	[137]
Chitosan-based bioactive films incorporating quercetin-loaded chitosan NPs (QCNPs)	Thickness: 43.1–45.6 μm. Intact morphology	-Enhanced thermal, mechanical, water vapor barrier, UV-light barrier, and radical scavenging properties. -Significant antibacterial activity.	[138]
Vaccarin-chitosan NPs f	Diameter: ~216.6 ± 10.1 nm Spherical-like morphology Zeta potential: +37.1 ± 1.2	-Increased cell migration with administration. -Improved and faster wound-healing effects on rat model, with complete recovery following 10 days of treatment.	[139]
Melatonin-loaded lecithin-chitosan NPs	Size: ~160.43 ± 4.45 nm Spherical and subspherical morphology Zeta potential: 25.0 ± 0.57 mV	-Accelerated wound healing and fibroblast proliferation on rat model.	[140]
Curcumin-loaded chitosan NPs containing hydrogels	Size: ~370 nm Spherical morphology Zeta potential: 41.4 mV	-Concentration-dependent antibacterial activity. -High biocompatibility. -Accelerated wound closure at day 14 compared to the control group.	[141]
Quercetin-loaded alginate/chitosan NPs	Spherical morphology Encapsulation efficiency up to 82.4% Loading capacity up to 46.5%	-Antibacterial activity of unloaded alginate/chitosan NPs with average ZOI values. -Notable ZOI value with sole administration of quercetin. -Significantly higher ZOI values with drug loading.	[142]
Licoricidin-loaded chitosan NPs	Size: ~90 nm Spherical morphology Zeta potential: >45 mV	-2-fold higher reduction in MIC values and complete inhibition of bacterial growth. -Prolonged inhibitory activity for 16 h, compared to drug alone (10 h)	[143]
Drug-loaded alginate-chitosan NPs	Size: ~100 nm Spherical and elliptical morphology Zeta potential: ~ −16.12 ± 3.06 mV	-Significant bacterial reduction in multiple strains. -Large ZOI by >10 mm.	[144]
Encapsulated alginate-chitosan NPs	Size: ~335.3 nm Spherical morphology Zeta potential: 45.1 ± 1 mV	-Significant reduction in MIC and MBC values with drug loading. -Anti-biofilm activity up to 65–80%.	[145]
Encapsulation of cellulose nanocrystals stabilized lysozymes in chitosan NPs	Size: 171.43–308.53 nm Spherical morphology Zeta potential: 9.21 mV–51.24 mV	-Significant antibacterial activity against *S. aureus* and *Vibrio parahaemolyticus*. -Reduced MIC values with increased particle size. -Reduced MBC values with changes in particle sizes.	[146]
Lemongrass essential oil-encapsulated chitosan NPs	Size: ~200 Spherical morphology Zeta potential: 36.3 mV	-Significant reduction in MIC values with drug loading. -Significant increase in ZOI with drug loading.	[147]
Chitosan hydrogels with activated/non-activated carbon NPs	Increased crystallinity index	-Effective absorption of heavy metals with a stronger affinity towards Pb. -High bactericidal activity -Loss of antibacterial activity following functionalization with carbon NPs due to lack of free positive charges.	[148]
Hybrid chitosan-silver NP-based films	-	-High mechanical stability. -Antibacterial activity Against *E. coli* with 0.5 cm Inhibition Zone	[149]
Chitosan NP-incorporated orthodontic micro-implants	Size: 70–100 nm	-Strong antibacterial activity by inhibition zones between 13–18.3 mm various strains -Significantly low MIC and MBC values of 8–16 µg/mL and 4–8.1 µg/mL, respectively.	[150]
Chitosan-based nanocomposite-coated titanium dental implants	Size: 26–52 nm Low coating coverage and larger size	-High ZOI range against various oral microorganisms. -Significant bactericidal effect.	[151]
Doxycycline-loaded chitosan NPs	Size: ~203.1 nm Spherical morphology Zeta potential: ~+32.3 mV	-Significant bacteriostatic activity, leading to complete inhibition of bacterial colonies.	[152]
Drug-loaded chitosan NPs	Size: 60.66–87.44 nm Spherical morphology Zeta potential: −15.26–−29.52 mV	-Controlled drug release. -Strong inhibitory effect with a high inhibition zone.	[153]

## Data Availability

Not applicable.
